# Whole Lotta Lipids—From HCV RNA Replication to the Mature Viral Particle

**DOI:** 10.3390/ijms21082888

**Published:** 2020-04-21

**Authors:** Hanna Bley, Anja Schöbel, Eva Herker

**Affiliations:** Institute of Virology, Philipps-University Marburg, 35043 Marburg, Germany; hanna.bley@uni-marburg.de (H.B.); anja.schoebel@uni-marburg.de (A.S.)

**Keywords:** hepatitis C virus (HCV), lipids, fatty acid, lipid remodeling, membrane vesicles, lipid droplets, lipid transfer proteins, HCV replication, HCV assembly, HCV egress

## Abstract

Replication of the hepatitis C virus (HCV) strongly relies on various lipid metabolic processes in different steps of the viral life cycle. In general, HCV changes the cells’ lipidomic profile by differentially regulating key pathways of lipid synthesis, remodeling, and utilization. In this review, we sum up the latest data mainly from the past five years, emphasizing the role of lipids in HCV RNA replication, assembly, and egress. In detail, we highlight changes in the fatty acid content as well as alterations of the membrane lipid composition during replication vesicle formation. We address the role of lipid droplets as a lipid provider during replication and as an essential hub for HCV assembly. Finally, we depict different ideas of HCV maturation and egress including lipoprotein association and potential secretory routes.

## 1. Fatty Acids in HCV Replication

**Figure 1:**
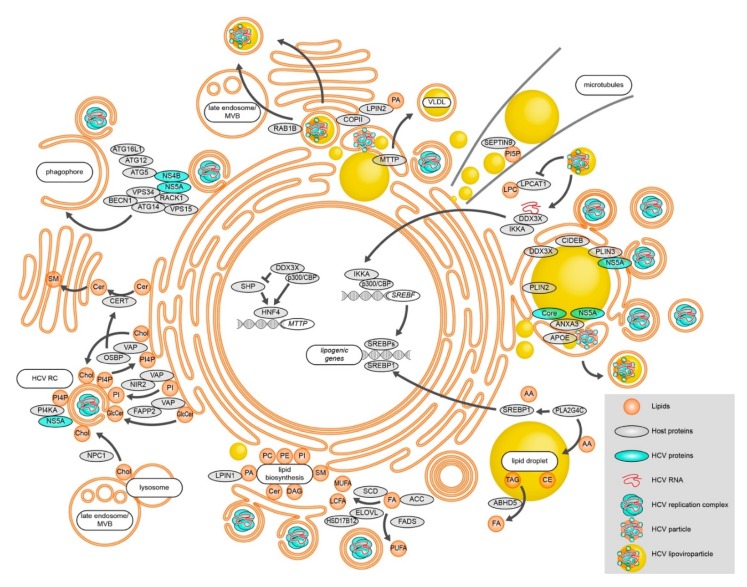
Modulation of lipid metabolic processes during HCV RNA replication, assembly, and egress. HCV alters cellular FA levels and changes lipid biosynthesis. Lipid transfer proteins are utilized to change the membrane lipid composition in order to efficiently establish vesicular replication compartments. Besides replication vesicles, phagophore membranes also support HCV RNA replication, connecting autophagy to HCV RNA replication. Different lipid-modulating host proteins localize to HCV assembly sites in close proximity to LDs and shape the emerging virions. HCV particles exit the cell at least partially via the canonical secretory pathway. Alternatively, secretion occurs via late endosomes/MVBs. See text for details.

Apart from being essential building blocks for cellular lipids, fatty acids (FAs) are involved in cellular signaling pathways and posttranslational protein modification. Concordant with other members of the *Flaviviridae* family, FAs play a role in HCV replication [1] (Figure 1). A recently published study combining metabolomics, transcriptomics, and proteomics to analyze HCV-induced changes in cellular pathways observed an accumulation of very-long chain fatty acids (VLCFAs) in Jc1-infected differentiated Huh7.5.1 cells [2]. Concurrently, peroxisomal function was impaired, indicating a decreased β-oxidation of VLCFAs in HCV-infected cells. A second lipidomic analysis comparing the lipid composition of Jc1-infected and uninfected Huh7.5 cells revealed an HCV-induced higher relative abundance of longer fatty acyl chains in triglycerides (TAG) and phosphatidylcholines (PC) as well as the increased incorporation of C18 FAs, especially monounsaturated oleic acid [3]. Additionally, Jc1 infection leads to elevated levels of free polyunsaturated fatty acids (PUFAs), namely, arachidonic acid (AA) (20:4n6), docosahexaenoic acid (22:6n3), and eicosapentaenoic acid (20:5n3). Likewise, the infection of Huh7.5 cells with JFH1 alters the FA composition of cellular lipids towards an augmented utilization of long fatty acyl chains and increased PUFA levels [4]. Depletion of different FA elongases (elongation of very long chain fatty acids protein, ELOVL) as well as the ∆9 desaturases (stearoyl-CoA desaturase, SCD, and its homologue SCD5) impairs viral RNA replication, whereas the ∆6 desaturase (acyl-CoA 6-desaturase, FADS2), the rate-limiting enzyme in PUFA synthesis, is primarily involved in virion production, suggesting a role for unsaturated fatty acids (UFAs) in HCV morphogenesis [3]. JFH1-infected cells also display higher C18:0/C16:0 and C20:4/C18:2 ratios, correlating with increased elongase and ∆6 desaturase activities [4]. Although HCV relies on an intact FA metabolism and increased cellular PUFA levels, treatment of Jc1-infected cells with FAs, especially PUFAs, decreases viral replication and subsequent virus production, presumably by distorting the ultrastructure of the membranous web that consists of single and double membrane vesicles harboring the HCV replicase complex [3]. Generation of FAs occurs via de novo lipogenesis, lipolysis or uptake from extracellular sources. Modulation and requirement of lipogenic pathways in HCV infection have been described earlier [5]. Adding up to this, inhibition or depletion of acetyl-CoA carboxylase (ACC), the enzyme that catalyzes malonyl-CoA synthesis from acetyl-CoA, results in reduced intracellular HCV RNA levels and consequently decreases virion production [6]. Additionally, ACC inhibition leads to a loss of cellular lipid droplets (LDs) and extensive changes in the host cell lipidome. While supplementation with free FAs restores LDs, neither HCV RNA levels nor infectious particle production is rescued, indicating that HCV relies on de novo lipogenesis to establish infection, and newly synthesized as well as exogenously derived free FAs are differentially metabolized during infection. On the contrary, infection with JFH1 causes oxidative stress-induced activation of AMP-activated protein kinase (AMPK), leading to increased phosphorylation and thereby inactivation of ACC. As a result, malonyl-CoA levels and de novo lipogenesis are reduced in JFH1-infected cells, while β-oxidation is enhanced, accompanied by an increase in nuclear peroxisome proliferator-activated receptor α (PPARα) and upregulation of β-oxidation genes [4]. In contrast, PPARα protein levels are reduced in Jc1-infected differentiated Huh7.5.1 cells, leading to decreased peroxisomal gene expression [2]. These contradictory results could indicate that Jc1 and JFH1 variably affect or usurp metabolic pathways, but using differentiated and thus non-dividing Huh7.5.1 cells versus Huh7.5 cells might also contribute to the divergent results. As mentioned above, (V)LCFAs are more abundantly present in HCV-infected cells [2,3,4]. Most recently, the 17-betahydroxysteroid dehydrogenase type 12 (HSD17B12), catalyzing the second elongation step in VLCFA synthesis, was identified as an important host factor for HCV replication [7]. HCV RNA replication in subgenomic replicon cells and viral particle production from JFH1-transfected cells are significantly impaired in HSD17B12-depleted cells, accompanied by a strong decrease in cytoplasmic LDs, reduced TAG and phosphatidylethanolamine (PE) levels, and a decreased oleic acid:palmitic acid ratio. Treatment with oleic acid restores LDs and partially rescues HCV RNA replication as well as particle production, indicating that FAs with acyl chains ≥C18 and VLCFA synthesis are important for efficient replication. Interestingly, Dengue (DENV) and Zika virus (ZIKV) production also require HSD17B12, highlighting the important role of FA metabolism in flavivirus replication [7]. Among other FA-modulating enzymes, several phospholipases have been identified as host factors for HCV [8,9,10,11,12]. The enzymatic activity of cytosolic phospholipase A2 gamma (PLA2G4C) is crucial for LD formation, and this function directly correlates with its requirement for HCV replication [12]. Treatment with arachidonic acid partially rescues LD levels in PLA2G4C-depleted cells, suggesting that free FAs generated by phospholipid hydrolysis promote LD formation. Additionally, PLA2G4C is upregulated in HCV-infected cells [10]. Following increased PLA2G4C expression, sterol regulatory element-binding protein (SREBP-1c) levels as well as the global cellular lipid content are elevated, but specific lipid composition is unaffected, indicating a role of PLA2G4C in HCV-induced lipogenesis [12]. 

## 2. HCV-Induced Membrane Remodeling

Membrane alterations are prominent features of cells that replicate the HCV RNA. Membrane lipid composition determines the membrane flexibility and fluidity, influencing the formation of the vesicular HCV replication organelles, the membranous web. Both aspects depend on the saturation grade of FAs, the acyl chain length, and the amount of sterols [1]. Lipids differentially shape membranes, contributing to the distinct curvatures observed in HCV-induced membrane remodeling. Whereas cholesterol (Chol), ceramides (Cer), UFAs, and PE are cone-shaped lipids, which lead to a negative curvature, phosphatidylinositol 4-phosphate (PI4P) and lysophospholipids (LysoPL) are inverted cone-shaped lipids, inducing positive curvature. Phosphatidylcholine (PC), phosphatidylinositol (PI), phosphatidic acid (PA), and sphingomyelin (SM) are of cylindrical shape and thus form a rather flat membrane [1]. A recent study showed that membrane lipids, especially cholesterol and PC, accumulate in the microsomal fraction during HCV infection [3]. In addition to HCV, other positive-strand RNA viruses, such as poliovirus and brome mosaic virus (BMV), display an enrichment of PC at the replication sites [13]. This indicates that positive-strand RNA viruses share similar elements to promote PC synthesis and recruitment to their replication complexes. To maintain phospholipid homeostasis and triglyceride biosynthesis, the conversion of PA to diacylglycerol (DAG) by the phosphatidate phosphatase Lipin (LPIN) is crucial. Humans possess three LPIN proteins: LPIN1, LPIN2, and LPIN3. While LPIN3 is not very well studied, LPIN1 and LPIN2 compensate for each other to maintain the lipid metabolism in the liver of knockout mice [14]. In contrast, distinct roles of LPIN1 and LPIN2 during HCV infection were described recently [15,16]. LPIN1 levels increase during HCV infection and its phosphatase activity is required for replication complex formation [16]. Thus, in the absence of LPIN1, membrane alterations in HCV-infected cells are less pronounced, leading to reduced intracellular viral RNA and extracellular titers. However, LPIN1 depletion has no effect on established HCV infections. On the other hand, LPIN2 silencing disturbs viral secretion without drastically changing HCV replication and assembly [15]. Mechanistically, knockdown of LPIN2 leads to fragmentation of the Golgi apparatus as well as elongation of mitochondria, indicating that LPIN2 function is crucial for intact organelle morphology. Interestingly, LPIN2 but not LPIN1 deficiency decreases the spread of both ZIKV and DENV. This highlights the dependency of flaviviruses on lipid metabolic pathways and membrane lipid remodeling. Another lipid class, sphingomyelins (SM), are present in the HCV virion and at microdomains of the replication complex [17]. HCV infection promotes SM synthesis by upregulation of sphingomyelin synthases 1 and 2, and inhibition of SM synthesis decreases HCV replication [17]. Functionally, SM binds and activates the non-structural HCV protein NS5B to attach the template RNA [18]. Additionally, it was shown that a cell culture-adapted version of JFH1, that generally promotes HCV particle production and increases infectivity compared to the wildtype, reduces SM secretion, likely due to a decreased SM content in the virions [19]. This is dependent on the interaction between NS5B and p7 carrying specific cell culture-adapted mutations, in the case of NS5B within the SM-binding pocket. These data underline the importance of the interaction between viral proteins and host lipids to regulate viral infectivity.

## 3. Another Brick in the Wall—Cholesterol in HCV Replication

In the past decade, a critical role for cholesterol in HCV replication was uncovered. Cholesterol-rich membrane domains have been described as pivotal for entry, replication, and infectivity of progeny virions, with a particular structural role in the replication vesicle membrane [18]. Further, cholesterol accumulation is observed in the perinuclear region in HCV-infected cells [3]. Indeed, cholesterol is delivered to and accumulated at vesicular replication organelles during HCV infection [20]. This study also described the HCV replication organelle as a dynamic complex by distinguishing between “old” and “new” NS5A positive foci. The “old” NS5A-positive membranous structures are the main target for cholesterol delivery and associate with LDs and the HCV core protein, whereas the “new” structures are formed de novo at distinct sites that facilitate the interaction between NS5A and the phosphatidylinositol 4-kinase (PI4KA) as well as the oxysterol-binding protein (OSBP) that both play crucial roles for efficient double membrane vesicle (DMV) formation during HCV infection (for details see below). 

## 4. Lipid Transfer Proteins

Upon HCV replication, NS5A binds and activates PI4KA, consequently increasing PI4P levels in the membranes. In the absence of PI4KA, NS5A accumulates at membranes, and the DMVs formed are smaller in diameter [5]. PI4P might promote positive membrane curvature due to its negatively charged headgroup, but also recruits the host proteins OSBP and phosphatidylinositol four-phosphate adaptor protein 2 (FAPP2) to PI4P rich domains. Both OSBP and FAPP2 are lipid transfer proteins (LTP) that bind PI4P as well as vesicle-associated membrane protein-associated proteins (VAP) anchored at the ER membrane. LTPs establish membrane contact sites (MCS) and enable lipid exchange between organelles [21]. OSBP and FAPP2 induce sterol and glycosphingolipid trafficking, respectively, to the replication organelles. OSBP additionally regulates the ceramide transfer protein (CERT), which organizes the flux of ceramides from the ER to the Golgi apparatus, where they are converted into sphingolipids. Inhibition of these proteins changes membranous web morphology, lipid composition, and consequently reduces HCV replication [20,22]. The PI transfer protein NIR2 (Pyk2 N-terminal domain-interacting receptor 2, also known as membrane-associated phosphatidylinositol transfer protein 1, PITPNM1) interacts with VAPs and is located in close proximity to NS5A-containing replication complexes [23]. NIR2 acts to restore PI levels at the replication sites to maintain elevated levels of PI4P, which is exchanged to cholesterol by OSBP [23]. This would support a consistent lipid flow between ER and the HCV replication organelles to enable continuous HCV replication. Another LTP residing at the late endosome and lysosomal membranes, the Niemann-Pick-type C1 (NPC1), is responsible for cholesterol transport to the membranous web [24]. Knockdown or inhibition of NPC1 induces cholesterol entrapment in lysosomal vesicles, lowers cholesterol levels at the replication sites, and disrupts membrane integrity. Treatment with the cholesterol transport inhibitor U18666A that directly inhibits NPC1 and consequently the formation of late endosome/multivesicular bodies (MVB), leads to an accumulation of virions in exosomes and blocks viral release, while not affecting RNA replication and HCV assembly [25]. However, the entrapped particles are lower in density, indicating an altered lipid composition. This illustrates the importance of the lipid content for fully infectious viral particle assembly and secretion. Thus, HCV depends on LTPs to provide a dynamic lipid supply to the replication organelle and to secure efficient replication, assembly, and secretion.

## 5. Autophagosomes, Membranes, and HCV RNA Replication

While there is consistent agreement that autophagy is critical for HCV replication, the detailed mechanism of how viral replication is tied to the cellular recycling machinery remains to be elucidated [26]. Recent studies have focused on a possible connection of HCV replication compartments and viral RNA replication to autophagosomes [27,28]. Proteins associated with membrane microdomains such as caveolin-1 are associated with autophagosomes in GFP-LC3-expressing cells harboring an HCV subgenomic replicon, but not with starvation-induced autophagosomes [28]. In addition, cholesterol is redistributed to HCV- but not to rapamycin-induced autophagosomes, and purified autophagosomes support HCV RNA replication in vitro. Removal of cholesterol by methyl-β-cyclodextrin treatment abolishes viral RNA replication in these purified autophagosomes, suggesting an HCV-specific association of membrane microdomains with autophagosomes that is required for HCV RNA replication. Autophagy starts with the formation of an isolation membrane, also known as a phagophore [29]. These crescent membrane structures support HCV RNA replication in vitro, and inhibition of phagophore maturation by depletion of syntaxin-7 (STX7) does not affect viral RNA levels [27]. Thus, effective HCV RNA replication does not necessarily require autophagosome maturation. The autophagy-related proteins ATG5-12/16L1 autophagosome elongation complex, but not LC3, associates with purified membranous web fractions from HCV replicon cells, and efficient HCV RNA replication depends on ATG5-12/16L1 complex formation [30]. Depletion of ATG7, an essential factor for ATG5-ATG12 conjugation and LC3 lipidation, significantly reduces the size of HCV-induced DMVs. In addition, cells lacking ATG12 also show a significant loss of DMVs, and remaining vesicles are smaller, whereas depletion of LC3 does not alter membrane vesicle structures, connecting the formation of HCV replication organelles to the nascent autophagosomal membranes rather than to mature autophagosomes. The non-structural HCV proteins NS4B and NS5A, fundamental contributors to DMV formation [18], also induce autophagy [26,31]. In this context, NS5A was recently identified to interact with the receptor for activated protein C kinase 1 (RACK1) and with proteins of the Beclin 1 (BECN1)-ATG14-VPS34-VPS15 complex that is essential for autophagy initiation [32]. The NS5A-ATG14 interaction is bridged by RACK1, which is a key adaptor for the formation of the BECN1-ATG14-VPS34-VPS15 complex upon starvation [33]. Both RACK1 and ATG14L are required for viral RNA replication as well as NS5A-induced autophagy. Depletion of RACK1 and ATG14 significantly reduces the number of DMVs, illustrating a role for autophagy initiation and thus autophagic membranes as origins of or contributors to the HCV replication organelles [32]. 

## 6. Come Together—LDs Provide a Stage for HCV Assembly

Since LDs were first proposed as essential organelles for HCV replication, plenty of research has been done to elucidate the detailed mechanism of how HCV exploits and relies on these lipid storage organelles [21]. The localization of core and NS5A to LDs is crucial for HCV assembly, and both proteins require diacylglycerol acyltransferase 1 (DGAT1) to traffic to LDs [34]. LDs mainly consist of a neutral lipid core (TAGs and cholesterol ester (CE)) surrounded by an amphipathic phospholipid monolayer that is decorated with different LD-associated proteins, including proteins of the perilipin (PLIN) family [35]. PLIN3 (also known as tail interacting protein of 47 kDa, TIP47) interacts with NS5A and has already been described as an essential host factor for HCV RNA replication and/or assembly [21]. Our group recently identified PLIN2 (also known as adipose differentiation-related protein, ADRP or adipophilin, ADFP), the major LD-coating protein in hepatocytes [36,37], as important factor for HCV morphogenesis [38]. In PLIN2-depleted cells, LDs and HCV replication organelles are enclosed by ER-derived double membranes. Due to these ultrastructural alterations, trafficking of core and NS5A to LDs and subsequent infectious viral particle formation are compromised. While LD homeostasis is still intact, a lack of PLIN2 slightly increases LD size and causes an enhanced lipolysis activity as well as β-oxidation, indicating differences in lipid flux. Of note, previous publications discussing the role of PLIN2 in HCV had inconsistent findings regarding LD morphology and viral replication [39,40]. Latest high-resolution imaging studies investigating the ultrastructure of HCV replication sites observed ER-wrapped LDs with DMV structures connected to the respective ER membranes [41]. Although HCV RNA replication already induces membrane-wrapped LDs, co-expression of the structural proteins significantly increases their number, revealing a close spatial coupling of assembly sites and replication vesicles in HCV infection. Besides membrane rearrangements, HCV-infected cells display many other structural changes. In JFH1-infected cells, the cytoskeleton-associated GTPase SEPTIN9 shows an increased filamentous distribution that is concurrent with the accumulation of microtubule filaments [42]. SEPTIN9 positively regulates HCV replication and is involved in the perinuclear accumulation of LDs observed in HCV-infected cells by interacting with phosphatidylinositol 5-phosphate (PI5P) and microtubules. Depletion of SEPTIN9 reduces LD number and size and decreases the TAG/DAG ratio, connecting its proviral role for HCV to its function in LD biogenesis. While DENV initiates lipolysis of LDs to generate free FAs [43], HCV has mainly been described to rely on SREBP-induced lipogenesis as a free FA source [18]. However, the adipose triglyceride lipase (ATGL)-cofactor ABHD5 (α/β hydrolase domain containing protein 5, also known as CGI-58), an LD-associated protein, was recently shown to be important for HCV assembly and egress, likely by being involved in HCV-induced lipid mobilization from LDs [44]. Mutations in ABHD5 can cause the Chanarin-Dorman syndrome, a lipid storage disorder in humans [45]. These ABHD5 Chanarin-Dorfman mutants do not localize to LDs or support LD lipolysis or HCV replication, suggesting that the proviral function of ABHD5 depends on its lipase cofactor function. Thus, apart from providing an assembly platform, LDs are usurped as an intracellular lipid source during HCV infection.

## 7. Dress for Success—HCV Lipoviroparticle Formation and Egress

Mature HCV virions are associated with lipoproteins, forming the so-called lipoviroparticle (LVP), and intact very-low-density-lipoprotein (VLDL) synthesis and the apolipoproteins APOE and APOB are crucial for HCV particle production in vitro [46]. Efficient virion assembly and infectivity depend on the direct interaction between the viral non-structural protein NS5A as well as the envelope protein E2 and APOE, thus incorporating APOE into the lipoviroparticle [21]. The calcium-dependent phospholipid-binding protein Annexin A3 (ANXA3) has been described as a critical cellular factor that relocates to LDs in infected cells and initiates the interaction between APOE and E2 [47]. Knockdown of ANXA3 lowers virion production and infectivity with a higher density of secreted particles. Cell-death-inducing DFFA-like effector B (CIDEB), an ER- and LD-associated protein and a modulator of the VLDL pathway [48,49], is entangled in the interaction of NS5A and APOE during HCV assembly [50]. The exact involvement of APOE in virus production is HCV strain and cell type specific [51]. However, most HCV strains prefer APOE incorporation to other apolipoproteins (APOA1, APOA2, APOC1, APOC3). Other lipid-modulating host proteins that associate with viral particles have been identified. It was previously reported that the PI(3,5)P_2_ 5-phosphatase FIG4 is associated with viral particles [52]. Additionally, FIG4 deficiency impairs CE levels and HCV infectivity, whereas overexpression has the opposite effect, both without changing HCV RNA levels. This indicates that FIG4 regulates virion formation in a CE-dependent manner. Interestingly, HCV-mediated downregulation of the ATP-dependent RNA helicase DDX3X decreases expression of the microsomal triglyceride transfer protein (MTTP) that is required for APOB lipidation during VLDL synthesis. Consequently, this initiates lipid accumulation and reduces APOB secretion in DDX3X-deficient cells [53]. DDX3X interacts with the CREB-binding protein-histone acetyltransferase (CBP) p300 (CBP/p300) to enhance acetylation of hepatocyte nuclear factor 4 (HNF4), which enhances its promotor binding affinity to the MTTP promotor and regulates VLDL secretion. In detail, DDX3X directly interacts with both HNF4 and small heterodimer partner (SHP), that positively or negatively modulate MTTP promotor activity, respectively. DDX3X disrupts the SHP/HNF4 heterodimer to promote HNF4 homodimer formation, thus inducing MTTP gene expression. The late stages of HCV replication are not yet fully elucidated, and recent publications disagree on the role of the canonical secretory lipoprotein pathway as an exit route for HCV particles. Together with APOE and APOB, a fraction of infectious HCV particles is detectable in COPII vesicles, the transport vesicles that facilitate the anterograde transport from the ER to the Golgi apparatus, arguing that LVPs are already formed in the ER [54]. Depletion of CIDEB, Sortilin, and KLHL12, proteins involved in VLDL secretion, significantly increases intracellular HCV titers and impairs HCV secretion, but the effect is only marginal compared to the strong decrease in APOB and APOE secretion. Proteins of the Rab GTPase family are regulators of vesicle trafficking. RAB1B, a major modulator of ER-to-Golgi transport, differentially regulates the secretion of APOE and APOB; whereas both apolipoproteins are secreted in a RAB1B-dependent manner, only APOB initially relies on RAB1B to exit the ER. Equally to APOB and in contrast to APOE, blocking RAB1B-mediated cargo exit from the ER impairs HCV egress and leads to an intracellular accumulation of infectious particles. Thus, HCV shares the early canonical secretory pathway with nascent VLDL [55]. Differential expression of lipid-modulating enzymes is a common characteristic of HCV-infected cells. The lyso-PC acyltransferase 1 (LPCAT1), a PC synthesizing enzyme that also localizes to LDs [56], is downregulated upon HCV infection, and LPCAT1 depletion revealed an increased specific infectivity of HCV particles that correlates with a lower buoyant density [57]. LPCAT1-deficient cells have fewer but larger LDs and elevated TAG levels, accompanied by an increased secretion of APOB and slightly elevated extracellular TAGs. If HCV egress is at least partially coupled to VLDL secretion, HCV might stimulate its own egress by decreasing LPCAT1 expression. On the contrary, VLDL components might be essential for infectious particle formation, but secretion pathways are divergent, since Rab GTPases and other key proteins involved in trans-Golgi-network (TGN)-endosomal trafficking are crucial for HCV egress, but are not required for lipoprotein secretion [58]. Further, HCV infection causes a redistribution of the TGN to core-decorated LDs, thereby spatially coupling HCV replication sites to the potential egress pathway. On the other hand, there are studies suggesting that at least part of the assembled particles exit via the endosomal pathway [59,60]. Inhibition of late endosomes/MVB leads to accumulation of viral particles in exosomes and impairs HCV particle release, but not assembly [25]. Moreover, glycoproteins in secreted particles have an EndoH- sensitive glycosylation pattern, pointing to a non-canonical secretion due to the absence of glycan processing by Golgi-associated enzymes [59]. In conclusion, unravelling the detailed mechanism of HCV maturation and egress is a process still ongoing.

## 8. Conclusions

Taken together, the findings indicate that HCV profoundly changes the host cell lipidome and exploits the underlying cellular pathways to efficiently reshape cellular membranes for replication vesicle formation. Phagophores have been implicated to serve as replication platforms while cytosolic LDs not only represent virion assembly platforms but, in addition to lipogenesis, provide lipids to fuel HCV infection. In the future, comparative studies on key lipogenic vs. lipolytic enzymes could unveil the contribution of either pathway to HCV infection. Likewise, the dynamics and timing of lipid metabolic changes as well as concurrent membrane and LD remodeling need to be elucidated and verified in hepatocytes under physiological conditions. 

## Abbreviations

AA	Arachidonic acid
ABHD5	α/β hydrolase domain containing protein 5
ACC	Acetyl-CoA carboxylase
ADFP	Adipophilin
ADRP	Adipose differentiation-related protein
AMPK	AMP-activated protein kinase
ANXA3	Annexin A3
APO	Apoplipoprotein
ATG	Autophagy-related protein
ATG12	Ubiquitin-like protein ATG12
ATG14	Beclin 1-associated autophagy-related key regulator
ATG16L1	Autophagy-related protein 16-1
ATG5	Autophagy protein 5
ATG7	Ubiquitin-like modifier-activating enzyme ATG7
ATGL	Adipose triglyceride lipase
BECN1	Beclin-1
BMV	Brome mosaic virus
CBP	CREB-binding protein-histone acetyltransferase
CE	Cholesterol ester
Cer	Ceramide
CERT	Ceramide transfer protein
Chol	Cholesterol
CIDEB	Cell-death-inducing DFFA-like effector B
DAG	Diacylgylcerol
DDX3X	ATP-dependent RNA helicase
DENV	Dengue Virus
DGAT1	Diacylglycerol acyltransferase 1
DMV	Double membrane vesicle
ELOVL	Elongation of very long chain fatty acids protein
ER	Endoplasmic reticulum
FA	Fatty acid
FADS2	Acyl-CoA 6-desaturase
FAPP2	Phosphatidylinositol four-phosphate adaptor protein 2
FIG4	PI(3,5)P2 5-phosphatase
GlcCer	Glucosylceramide
HCV	Hepatitis C virus
HNF4	Hepatocyte nuclear factor 4
HSD17B12	17-betahydroxysteroid dehydrogenase type 12
IKKA	Inhibitor of nuclear factor kappa-B kinase subunit alpha
KLHL12	Kelch-like protein 12
LC3	Microtubule-associated proteins 1A/1B light chain 3B
LD	Lipid droplet
LPC	Lysophosphatidylcholine
LPCAT1	Lyso-PC acyltransferase 1
LPIN	Lipin
LTP	Lipid transfer protein
LVP	Lipoviroparticle
LysoPL	Lysophospholipid
MCS	Membrane contact sites
MTTP	Microsomal triglyceride transfer protein
MUFA	Monounsaturated fatty acid
MVB	Multivesicular bodies
NIR2	Pyk2 N-terminal domain-interacting receptor 2
NPC1	Niemann-Pick type C1
NS	Non-structural
OSBP	Oxysterol-binding protein
PA	Phosphatidic acid
PC	Phosphatidylcholine
PE	Phosphatidylethanolamine
PI	Phosphatidylinositol
PI4K	Phosphatidylinositol 4-kinase
PI4P	Phosphatidylinositol 4-phosphate
PI5P	Phosphatidylinositol 5-phosphate
PITPNM1	Membrane-associated phosphatidylinositol transfer protein 1
PLA2G4C	Cytosolic phospholipase A2 gamma
PLIN	Perilipin
PPARα	Peroxisome proliferator-activated receptor α
PUFA	Polyunsaturated fatty acid
RAB1B	Ras-related protein Rab-1B
RACK1	Receptor for activated protein C kinase 1
SCD	Stearoyl-CoA desaturase
SHP	Small heterodimer partner
SM	Sphingomyelin
SREPB1	Sterol regulatory element-binding protein
STX7	Syntaxin 7
TAG	Triglycerides
TGN	Trans-Golgi-network
TIP47	Tail interacting protein of 47 kDa
UFA	Unsaturated fatty acid
VAP	Vesicle-associated membrane protein-associated protein
(V)LCFA	(Very)-long chain fatty acid
VLDL	Very-low-density-lipoprotein
VPS15	Phosphoinositide 3-kinase regulatory subunit 4
VPS34	Phosphatidylinositol 3-kinase catalytic subunit type 3
ZIKV	Zika Virus
